# Association of oxidative balance score with hearing loss and tinnitus: NHANES 1999–2018

**DOI:** 10.3389/fnut.2024.1421605

**Published:** 2024-06-19

**Authors:** Haohong Lai, Jiyuan Yin, Haidi Yang

**Affiliations:** Department of Otolaryngology, Sun Yat-sen Memorial Hospital, Sun Yat-sen University, Guangzhou, Guangdong, China

**Keywords:** oxidative stress, oxidative balance score, antioxidant, hearing loss, pure-tone average, tinnitus

## Abstract

**Background:**

Oxidative stress is associated with the occurrence of hearing loss and tinnitus. The oxidative balance score (OBS), a composite indicator evaluating the balance between antioxidant and pro-oxidative components across various dietary and lifestyle factors, indicates the overall oxidative balance status. However, the association of OBS with hearing loss and tinnitus has not been reported previously.

**Methods:**

Cross-sectional data from the National Health and Nutrition Examination Survey (NHANES) 1999–2018 were analyzed. Weighted multivariable logistic regression, weighted multivariable linear regression, and restricted cubic spline curve (RCS) regression were employed to explore the relationship between OBS and hearing loss at speech, low, and high frequencies, along with tinnitus. Subgroup analysis and sensitivity analysis were used to ascertain the consistency across subgroups and stability of the results.

**Results:**

We included 13,715 and 21,644 individuals to investigate the association between OBS and hearing loss, as well as between OBS and tinnitus, respectively. The second, third, and fourth quartiles of OBS were significantly associated with a lower risk of hearing loss at speech, low, and high frequencies, as well as tinnitus, compared to the lowest quartile. The RCS regression analysis indicated a negative linear association of OBS with hearing loss and tinnitus. Most associations were maintained in subgroup analysis and sensitivity analysis. Additionally, the dietary and lifestyle OBS independently contribute to the protection against hearing loss and tinnitus.

**Conclusion:**

OBS is negatively correlated with the risk of hearing loss and tinnitus. The findings suggest that combined antioxidant diet and lifestyle hold promise as potential strategies for reducing the prevalence of hearing loss and tinnitus.

## 1 Introduction

Hearing loss is a prevalent otological disorder that affects a significant portion of the global population. Current estimates suggest that approximately 1.5 billion individuals are experiencing different degrees of hearing loss globally, and this figure is projected to increase to 2.4 billion by 2050 (1). Recognized as the fifth leading contributor to disability worldwide by the Global Burden of Disease Study (2), hearing loss exerts profound effects on individuals’ quality of life and psychological well-being (3–5). Regrettably, effective hearing restoration methods are lacking in the majority of cases (6). Hearing loss is the most common associated condition and the primary risk factor for tinnitus, which is defined as the perception of phantom sounds or noise without corresponding external stimuli. The increasing prevalence of hearing loss contributes to the growing incidence of tinnitus (7, 8). Currently, tinnitus affects an estimated 740 million individuals worldwide (9), imposing a high economic burden on society. However, due to the heterogeneity of the condition, there is scant evidence supporting the effectiveness of standard treatments for tinnitus (10). Therefore, effective interventions assume paramount importance for both hearing loss and tinnitus.

Previous research indicates that lifestyle elements like smoking, alcohol intake, and physical activity are linked with both hearing loss and tinnitus, presenting opportunities for possible interventions (10–15). Furthermore, dietary factors, as modifiable risk factors for hearing loss and tinnitus, have increasingly received attention in recent years. Single nutrients or dietary supplements (16–20), as well as dietary patterns (21–23), have demonstrated potential in protecting against hearing loss and tinnitus. Although the underlying mechanisms by which these lifestyle and dietary factors affect hearing loss and tinnitus remain incompletely understood, antioxidant properties are a primary focus of speculation (24, 25).

Increasing evidence suggests the pivotal involvement of oxidative stress (OS) in the pathophysiological mechanisms underlying hearing loss and tinnitus (26, 27). OS signifies the imbalance between reactive oxygen species (ROS) and the antioxidant defenses within cells (28). Excessive free radicals and ROS can cause DNA damage and lipid and protein degradation in tissues with high metabolic demands, such as the cochlea, leading to cell death (29, 30). Moreover, OS can trigger mutations in mitochondrial DNA, which disrupt mitochondrial function and cause cochlear cell apoptosis (31, 32). Moreover, antioxidants have demonstrated protective effects against hearing loss and tinnitus (24–26, 33), underscoring the potential significance of assessing the antioxidant effects of lifestyle and dietary factors for protecting against hearing loss and tinnitus.

The oxidative balance score (OBS), a tool evaluating the balance between antioxidant and pro-oxidative components across diverse dietary and lifestyle factors, has emerged as a promising metric (34). Recent studies have associated OBS with various diseases, such as depression (35), cardiovascular disease (36), kidney stones (37), and non-alcoholic fatty liver disease (38), demonstrating that a higher OBS is linked to a lower risk of these diseases. However, there are currently no studies on the correlation between OBS and hearing loss as well as tinnitus. To address this gap, our study investigates, for the first time, the association of OBS with hearing loss and tinnitus.

## 2 Materials and methods

### 2.1 Study population

**Figure 1:**
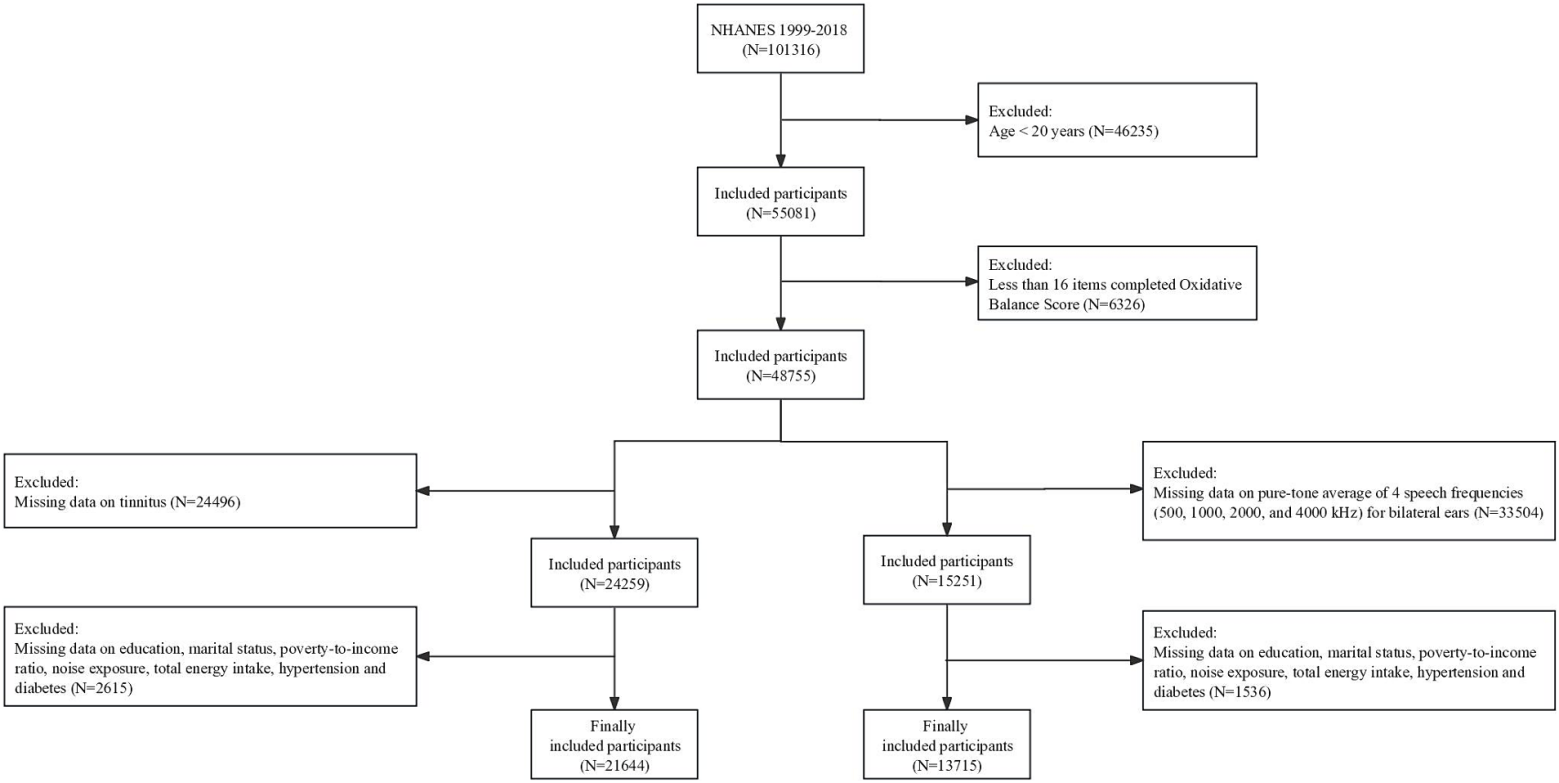
Flow chart of the selection strategy. NHANES, National Health and Nutrition Examination Survey.

We conducted a cross-sectional investigation using data from the National Health and Nutrition Examination Survey (NHANES). NHANES is a population-based survey employing complex, multistage, and probability sampling techniques to procure data representative of the U.S. population. The Research Ethics Reviewer Board of the National Center for Health Statistics (NCHS) approved the study protocol, and all participants provided written informed consent. Among 101,316 participants in the NHANES 1999–2018, we included two distinct study populations to investigate the association between OBS and hearing loss, as well as the association between OBS and tinnitus. Individuals were excluded if (1) they are less than 20 years old (*N* = 46,235), (2) they had less than 16 items among the total of 20 components of the OBS (*N* = 6,326), (3) they had missing data on the pure-tone average (PTA) of speech frequencies (0.5, 1, 2, and 4 kHz) for bilateral ears (*N* = 33,504) or missing data on tinnitus (*N* = 24,496), and (4) they had incomplete covariates data including educational level, marital status, family income-to-poverty ratio (PIR), noise exposure, total energy intake, hypertension, and diabetes (*N* = 1,536 in the study of hearing loss; *N* = 2,615 in the study of tinnitus). Ultimately, a total of 13,715 and 21,644 individuals were included in study population 1 and study population 2, respectively (Figure 1).

### 2.2 Oxidative balance score (exposure)

NHANES conducted nutritional assessments through in-person 24 h dietary recall interviews administered by trained dietary interviewers to participants of all age groups. The sample design and data Research Topic were overseen by the National Center for Health Statistics (NCHS), while the methodology for data Research Topic, as well as subsequent data processing and review, were managed by the USDA Food Survey Research Group (FSRG). The determination of OBS was based on earlier studies (34–39). OBS was computed through the evaluation of 16 dietary and 4 lifestyle components, comprising 15 antioxidants and 5 pro-oxidants. The total OBS score was calculated by adding up individual component scores, with a higher OBS value indicating increased exposure to antioxidants. The first dietary review interview provided data on the intake of 16 nutrients, which include dietary fiber, carotene, riboflavin, niacin, vitamin B_6_, total folate, vitamin B_12_, C, and E, calcium, magnesium, zinc, copper, selenium, total fat, and iron. The four lifestyle components comprised physical activity, alcohol consumption, smoking status, and body mass index (BMI). Physical activity was quantified by multiplying the metabolic equivalent (MET) score by the frequency and duration of each physical activity performed per week. Alcohol consumption was determined by calculating the mean number of alcoholic beverages consumed per day, regardless of the type of beverage. The intensity of smoking was gauged through cotinine levels, the primary metabolite of nicotine, serving as an indicator for both active smoking and exposure to secondhand smoke. BMI was calculated as weight in kilograms divided by height in meters squared. Among these components, total fat, iron, alcohol consumption, smoking status, and BMI were classified as pro-oxidants, while the remainder were categorized as antioxidants. Alcohol consumption was classified into three categories: non-drinkers, moderate drinkers (0–30 g/day for men and 0–15 g/day for women), and heavy drinkers (≥30 g/day for men and ≥ 15 g/day for women), with corresponding scores of 2, 1, and 0, respectively. Subsequently, the remaining components were stratified by gender and divided into three groups based on their tertiles, which were calculated after weighting. Antioxidants received scores ranging from 0 to 2 across the first to third tertiles, while pro-oxidants received scores ranging from 2 to 0, respectively (Supplementary Tables S1, S2). To minimize data loss and enhance data integrity and representativeness, participants with 16 or more complete data points out of the 20 OBS components were included. For any missing OBS components, regardless of their classification as antioxidants or pro-oxidants, a score of 0 was assigned (35).

### 2.3 Hearing loss and tinnitus (outcome)

The audiometry measurement of NHANES, which was pure-tone air conduction audiometry, was conducted by a highly trained examiner using audiometers with standard headphones and insert earphones in a calibrated sound booth. Participants underwent hearing threshold testing in both ears across seven frequencies (0.5, 1, 2, 3, 4, 6, and 8 kHz) over an intensity range from −10 to 120 dB. To ensure measurement reliability, the 1 kHz frequency was examined twice in each ear, with results excluded if a discrepancy exceeding 10 dB between the two measurements was observed. Further information on the audiometric measurement could be found in the Audiometry-Procedures-Manual of the NCHS. The PTA of speech frequencies (0.5, 1, 2, and 4 kHz) was calculated and defined as SF-PTA (speech-frequency pure-tone average). Additionally, the PTA of low frequencies (0.5, 1, and 2 kHz) is calculated and termed LF-PTA (low-frequency pure-tone average), while the PTA of high frequencies (3, 4, and 6 kHz) is defined as HF-PTA (high-frequency pure-tone average). All individuals underwent a comparison of their left and right PTA results, with the ear exhibiting better hearing chosen as the hearing index. According to the World Report on Hearing of the World Health Organization (40), PTA ≥ 20 dB is classified as hearing loss. Consequently, SF-PTA, LF-PTA, and HF-PTA ≥ 20 dB are, respectively, defined as speech-frequency hearing loss (SFHL), low-frequency hearing loss (LFHL), and high-frequency hearing loss (HFHL). The tinnitus occurrence (yes or no) of individuals was determined based on answers to the question, “In the past 12 months, have you been bothered by ringing, roaring, or buzzing in your ears or head that lasts for 5 min or more?”

### 2.4 Covariates

Sociodemographic data, including age, gender, race, educational level, marital status, and PIR, were collected through self-reported interviews. We combined Mexican American, other Hispanic, and other race into the category “Others” (41). The educational level was coded as high school graduate or less, college or above. Marital status was classified as married/living with partner, never married, and widowed/divorced/separated. PIR was grouped as *≤*130, >130 to 350%, and > 350%. In addition, risk factors closely correlated with hearing loss and tinnitus, such as noise exposure, hypertension, and diabetes (42–47), along with total energy intake, were included as covariates. The noise exposure (yes or no) of individuals depends on whether they are exposed to noise at work, after work, or from firearms. Any affirmative response to these exposures indicated the presence of noise exposure. Hypertension was characterized by meeting any of these criteria: (1) a history of hypertension; (2) taking antihypertensive medications; or (3) a mean blood pressure ≥ 140/90 mmHg during the physical examination. Diabetes was characterized by meeting any of these criteria: (1) a history of diabetes; (2) taking diabetic pills or the use of insulin injections; (3) a fasting blood glucose level ≥ 7.0 mmol/L; or (4) an HbA1c level ≥ 6.5%.

### 2.5 Statistical analysis

Descriptive data for baseline characteristics were presented as the weighted mean (standard errors) for continuous variables and sample numbers (weighted percentages) for categorical variables. Group differences were evaluated utilizing the *t*-test for continuous variables and the chi-square test for categorical variables. To ensure the robustness of our findings regarding the association between OBS and hearing loss, weighted multivariate logistic regression models and weighted multivariate linear regression models were simultaneously employed to investigate the associations between OBS and hearing loss (SFHL, LFHL, and HFHL), as well as the associations between OBS and PTA (SF-PTA, LF-PTA, and HF-PTA), respectively. As for tinnitus, weighted multivariate logistic regression models were used. Three models were implemented in the regression analyses. Model 1 was a crude model adjusted for age, gender, and race. Model 2 included additional adjustments for educational level, marital status, PIR, total energy intake, and noise exposure. Model 3 encompassed further adjustments for hypertension and diabetes, in addition to the variables in Model 2. Considering the potential for a nonlinear association between OBS and hearing loss as well as tinnitus, we also divided the continuous OBS into quartiles to form a categorical variable, and then calculated the *P* for trend. The OBS was further categorized into dietary OBS and lifestyle OBS to explore their associations with hearing loss as well as tinnitus independently. To ascertain the consistency of the results across subgroups and assess their stability, subgroup and sensitivity analyses were performed. We performed exploratory stratified analyses across all covariates to examine the associations between OBS and hearing loss as well as tinnitus across different population subgroups. Among these, we stratified individuals into elderly (≥60 years) and non-elderly (<60 years) groups to explore potential age-related differences in the association between OBS and auditory health outcomes. Additionally, multiplicative interaction tests were conducted to assess the potential interaction between covariates and OBS. In the sensitivity analyses, we divided the OBS by tertile or quintile and then analyzed the relationship between OBS and hearing loss as well as tinnitus using weighted multivariate logistic regression. Moreover, in order to address the potential selection bias due to excluding participants with missing data, we utilized multiple imputation methods to fill in missing covariates and recalculated the OBS. Subsequently, we analyzed the association between OBS and hearing loss as well as tinnitus in imputed data. Finally, restricted cubic spline (RCS) regression analysis was employed to illuminate the association of OBS with hearing loss as well as tinnitus.

Accounting for the complex sampling design of NHANES, sample weights were incorporated into all analyses. Statistical analyses were performed using R version 4.3.1. A significance level of *p* < 0.05 (two-tailed) was adopted for statistical significance.

## 3 Results

### 3.1 Baseline characteristics

Table 1 illustrates the baseline characteristics of two study populations. In the hearing loss study, 13,715 individuals were included with an average age of 46.53 years, among whom 3,455 were diagnosed with hearing loss. In the tinnitus study, 21,644 individuals were included with an average age of 47.43 years, among whom 4,706 were diagnosed with tinnitus. Both studies stratified participants into diseased and non-diseased groups. With the exception of total energy intake, which showed no significant difference between tinnitus and non-tinnitus patients, significant differences were observed in all covariates between the diseased and non-diseased groups. Participants diagnosed with hearing loss or tinnitus were more likely to be older, male, non-Hispanic white, divorced/separated/widowed. They also tended to have a lower educational level and family income, along with a history of noise exposure, hypertension, or diabetes. Importantly, participants with hearing loss or tinnitus tended to receive lower OBS.

**Table 1:** Weighted characteristics of the study populations.

Characteristics	Total	Non-HL	HL	*p* value	Total	Non-tinnitus	Tinnitus	*p* value
Total	*n* = 13,715	*n* = 10,260	*n* = 3,455		*n* = 21,644	*n* = 16,938	*n* = 4,706	
Age, year, Mean (SE)	46.53 (0.34)	41.95 (0.32)	63.55 (0.49)	<0.001	47.43 (0.27)	46.50 (0.28)	50.65 (0.38)	<0.001
Sex, *n* (%)				<0.001				<0.001
Male	6,730 (48.78)	4,633 (46.22)	2097 (58.30)		10,462 (47.97)	8,105 (47.11)	2,357 (50.99)	
Female	6,985 (51.22)	5,627 (53.78)	1,358 (41.70)		11,182 (52.03)	8,833 (52.89)	2,349 (49.01)	
Race, *n* (%)				<0.001				<0.001
Non-Hispanic White	6,080 (69.52)	4,084 (66.88)	1996 (79.32)		10,191 (70.43)	7,586 (68.67)	2,605 (76.59)	
Non-Hispanic Black	2,953 (10.74)	2,427 (11.73)	526 (7.07)		4,419 (10.74)	3,689 (11.48)	730 (8.13)	
Others	4,682 (19.74)	3,749 (21.39)	933 (13.61)		7,034 (18.83)	5,663 (19.85)	1,371 (15.28)	
Education level, *n* (%)				<0.001				<0.001
High school graduate or less	6,378 (37.53)	4,342 (34.27)	2036 (49.65)		10,926 (41.16)	8,298 (39.31)	2,628 (47.58)	
College or above	7,337 (62.47)	5,918 (65.73)	1,419 (50.35)		10,718 (58.84)	8,640 (60.69)	2078 (52.42)	
Marital status, *n* (%)				<0.001				<0.001
Married/living with a partner	8,366 (63.52)	6,262 (63.35)	2,104 (64.12)		13,151 (62.87)	10,365 (63.52)	2,786 (60.58)	
Divorced/separated/widowed	2,855 (18.01)	1703 (14.91)	1,152 (29.56)		4,885 (19.24)	3,621 (17.98)	1,264 (23.63)	
Never married	2,494 (18.47)	2,295 (21.74)	199 (6.32)		3,608 (17.89)	2,952 (18.50)	656 (15.79)	
Family PIR, *n* (%)				<0.001				<0.05
≤1.3	4,112 (21.99)	3,072 (22.38)	1,040 (20.55)		6,507 (22.44)	4,949 (21.86)	1,558 (24.44)	
1.3–3.5	5,263 (34.96)	3,750 (33.07)	1,513 (41.98)		8,436 (35.91)	6,631 (35.92)	1805 (35.87)	
>3.5	4,340 (43.05)	3,438 (44.55)	902 (37.47)		6,701 (41.65)	5,358 (42.22)	1,343 (39.69)	
Noise exposure, *n* (%)				<0.001				<0.001
No	6,731 (44.49)	5,270 (45.83)	1,461 (39.53)		12,037 (50.26)	9,844 (52.90)	2,193 (41.03)	
Yes	6,984 (55.51)	4,990 (54.17)	1994 (60.47)		9,607 (49.74)	7,094 (47.10)	2,513 (58.97)	
Hypertension, *n* (%)				<0.001				<0.001
No	8,235 (64.81)	6,990 (71.67)	1,245 (39.27)		12,766 (64.16)	10,419 (66.49)	2,347 (56.07)	
Yes	5,480 (35.19)	3,270 (28.33)	2,210 (60.73)		8,878 (35.84)	6,519 (33.51)	2,359 (43.93)	
Diabetes, *n* (%)				<0.001				<0.001
No	11,412 (87.09)	9,019 (90.67)	2,393 (73.63)		18,141 (87.75)	14,343 (88.27)	3,798 (85.96)	
Yes	2,303 (12.94)	1,241 (9.33)	1,062 (26.37)		3,503 (12.25)	2,595 (11.73)	908 (14.04)	
Total energy intake, Mean (SE)	2196.30 (10.99)	2235.81 (11.18)	2049.39 (24.86)	<0.001	2167.70 (9.08)	2158.72 (9.92)	2198.97 (22.96)	0.116
OBS, *n* (%)				<0.001				0.001
Q1	3,274 (21.71)	2,297 (20.49)	977 (26.27)		5,196 (21.61)	3,949 (20.88)	1,247 (24.18)	
Q2	3,501 (24.06)	2,567 (23.74)	934 (25.26)		5,511 (24.12)	4,304 (23.90)	1,207 (24.90)	
Q3	3,506 (26.28)	2,651 (26.29)	855 (26.22)		5,536 (26.28)	4,390 (26.80)	1,146 (24.45)	
Q4	3,434 (27.95)	2,745 (29.48)	689 (22.25)		5,401 (27.99)	4,295 (28.42)	1,106 (26.47)	

Values were presented as the weighted mean (standard errors) or sample numbers (weighted percentages). The differences between groups were assessed using the *t* test or the chi-square test (*p*-value < 0.05 indicates statistical significance). HL, hearing loss; PIR, income-to-poverty ratio; OBS, oxidative balance score; Q, quartile.

### 3.2 Association between OBS and hearing loss as well as tinnitus

As shown in Table 2, weighted multivariate logistic regression and weighted multivariate linear regression analyses demonstrated associations between OBS and both SFHL and tinnitus. In the fully adjusted model (Model 3), OBS exhibited an inverse correlation with both SFHL and tinnitus, with ORs (95% CI) of 0.969 (0.957 to 0.982) and 0.978 (0.971 to 0.986), respectively, both of which were statistically significant (*p* < 0.001). OBS also showed a negative correlation with the SF-PTA, with a β coefficient (95% CI) of −0.123 (−0.158 to −0.089, *p* < 0.001). In Model 3, the second, third, and fourth OBS quartiles all showed significantly negative associations with the risk of SFHL compared to the lowest quartile, with ORs (95% CI) of 0.709 (0.570 to 0.882, *p* = 0.002), 0.654 (0.511 to 0.838, *p* < 0.001), and 0.532 (0.415 to 0.681, *p* < 0.001), respectively. Regarding the association between OBS and SF-PTA, although the negative correlation between the second quartile of OBS and the SF-PTA did not reach significance compared to the lowest quartile (Q2: β = −0.642, −1.372 to 0.088, *p* = 0.084), outcomes for the third and highest quartiles resembled those observed for SFHL (Q3: β = −1.640, −2.333 to −0.947, *p* < 0.001; Q4: β = −2.177, −2.944 to −1.411, *p* < 0.001). For tinnitus, the second, third, and fourth OBS quartiles all displayed significantly negative associations with the risk of tinnitus compared to the first quartile, as evidenced by ORs (95% CI) of 0.862 (0.750 to 0.992, *p* < 0.05), 0.717 (0.625 to 0.823, *p* < 0.001), and 0.709 (0.604 to 0.833, *p* < 0.001), respectively. Trend tests for SFHL, SF-PTA, and tinnitus all indicated statistically significant decreasing trends (*p* for trend <0.001). Notably, the analysis results were relatively stable across different models.

**Table 2:** Association between OBS and hearing loss as well as tinnitus.

	Model 1		Model 2		Model 3	
	OR/β (95%CI)	*p* value	OR/β (95%CI)	*p* value	OR/β (95%CI)	*p* value
**SFHL**						
OBS (continuous)	0.970 (0.959, 0.980)	<0.001	0.968 (0.956, 0.980)	<0.001	0.969 (0.957, 0.982)	<0.001
OBS (quartile)						
Q1	Reference		Reference		Reference	
Q2	0.705 (0.573, 0.868)	0.001	0.702 (0.567, 0.868)	0.001	0.709 (0.570, 0.882)	0.002
Q3	0.657 (0.524, 0.823)	<0.001	0.646 (0.506, 0.825)	<0.001	0.654 (0.511, 0.838)	<0.001
Q4	0.527 (0.425, 0.652)	<0.001	0.516 (0.403, 0.661)	<0.001	0.532 (0.415, 0.681)	<0.001
p for trend	<0.001		<0.001		<0.001	
**SF-PTA**						
OBS (continuous)	−0.144 (−0.177, −0.111)	<0.001	−0.128 (−0.163, −0.094)	<0.001	−0.123 (−0.158, −0.089)	<0.001
OBS (quartile)						
Q1	Reference		Reference		Reference	
Q2	−0.917 (−1.639, −0.196)	<0.05	−0.694 (−1.410, 0.022)	0.057	−0.642 (−1.372, 0.088)	0.084
Q3	−1.983 (−2.600, −1.366)	<0.001	−1.700 (−2.387, −1.014)	<0.001	−1.640 (−2.333, −0.947)	<0.001
Q4	−2.685 (−3.385, −1.986)	<0.001	−2.277 (−3.041, −1.513)	<0.001	−2.177 (−2.944, −1.411)	<0.001
p for trend	<0.001		<0.001		<0.001	
**Tinnitus**						
OBS (continuous)	0.986 (0.979, 0.992)	<0.001	0.977 (0.970, 0.985)	<0.001	0.978 (0.971, 0.986)	<0.001
OBS (quartile)						
Q1	Reference		Reference		Reference	
Q2	0.882 (0.768, 1.011)	0.072	0.854 (0.743, 0.982)	<0.05	0.862 (0.750, 0.992)	<0.05
Q3	0.762 (0.660, 0.881)	<0.001	0.707 (0.616, 0.812)	<0.001	0.717 (0.625, 0.823)	<0.001
Q4	0.794 (0.694, 0.909)	<0.001	0.695 (0.592, 0.816)	<0.001	0.709 (0.604, 0.833)	<0.001
p for trend	<0.001		<0.001		<0.001	

Model 1: Adjusted for age, gender, and race.

Model 2: Combination of model 1 and educational level, marital status, PIR, noise exposure, and total energy intake.

Model 3: Combination of model 2 and hypertension, and diabetes.

OBS, oxidative balance score; Q, quartile; SFHL, speech-frequency hearing loss; SF-PTA, speech-frequency pure-tone average; OR, odds ratios; β, regression coefficient; CI, confidence interval; PIR, income-to-poverty ratio.

The association between OBS and both LFHL and HFHL is shown in Supplementary Table S3. The inverse association between OBS and both LFHL and HFHL was similar to that observed in the analysis of the speech-frequency group mentioned above, and the declining trend in both associations remained statistically significant. Additionally, the results indicate that the negative association between OBS and HFHL may be weaker compared to that between OBS and LFHL.

### 3.3 Association between dietary/lifestyle OBS and hearing loss as well as tinnitus

The association between dietary/lifestyle OBS and SFHL, SF-PTA, and tinnitus was investigated using weighted multivariate logistic regression analysis, with the results presented in Table 3. Upon adjustment for confounding variables, both dietary and lifestyle OBS were significantly linked to lower SFHL risk, with ORs (95% CI) of 0.972 (0.959 to 0.984, *p* < 0.001) for dietary OBS and 0.920 (0.873 to 0.970, *p* = 0.002) for lifestyle OBS. Similarly, both showed negative associations with SF-PTA, presenting β coefficients (95% CI) of −0.117 (−0.154 to −0.081, *p* < 0.001) for dietary OBS and − 0.302 (−0.458 to −0.146, *p* < 0.001) for lifestyle OBS. Moreover, both dietary and lifestyle OBS were significantly inversely related to tinnitus, with ORs (95% CI) of 0.981 (0.972 to 0.989, *p* < 0.001) and 0.936 (0.906 to 0.967, *p* < 0.001), respectively. In summary, both dietary and lifestyle OBS independently demonstrated negative associations with SFHL and tinnitus.

**Table 3:** Association between dietary/lifestyle OBS and hearing loss as well as tinnitus.

	Model 1		Model 2		Model 3	
	OR/β (95%CI)	*p* value	OR/β (95%CI)	*p* value	OR/β (95%CI)	*p* value
**SFHL**						
Dietary OBS	0.974 (0.963, 0.984)	<0.001	0.971 (0.958, 0.984)	<0.001	0.972 (0.959, 0.984)	<0.001
Lifestyle OBS	0.869 (0.824, 0.915)	<0.001	0.910 (0.864, 0.958)	<0.001	0.920 (0.873, 0.970)	0.002
**SF-PTA**						
Dietary OBS	−0.129 (−0.161, −0.096)	<0.001	−0.119 (−0.156, −0.082)	<0.001	−0.117 (−0.154, −0.081)	<0.001
Lifestyle OBS	−0.558 (−0.724, −0.392)	<0.001	−0.342 (−0.493, −0.192)	<0.001	−0.302 (−0.458, −0.146)	<0.001
**Tinnitus**						
Dietary OBS	0.990 (0.983, 0.997)	0.005	0.980 (0.972, 0.989)	<0.001	0.981 (0.972, 0.989)	<0.001
Lifestyle OBS	0.907 (0.877, 0.938)	<0.001	0.930 (0.901, 0.961)	<0.001	0.936 (0.906, 0.967)	<0.001

Model 1: Adjusted for age, gender, and race.

Model 2: Combination of model 1 and educational level, marital status, PIR, noise exposure, and total energy intake.

Model 3: Combination of model 2 and hypertension, and diabetes.

OBS, oxidative balance score; SFHL, speech-frequency hearing loss; SF-PTA, speech-frequency pure-tone average; OR, odds ratios; β, regression coefficient; CI, confidence interval; PIR, income-to-poverty ratio.

### 3.4 Subgroup analysis

**Figure 2:**
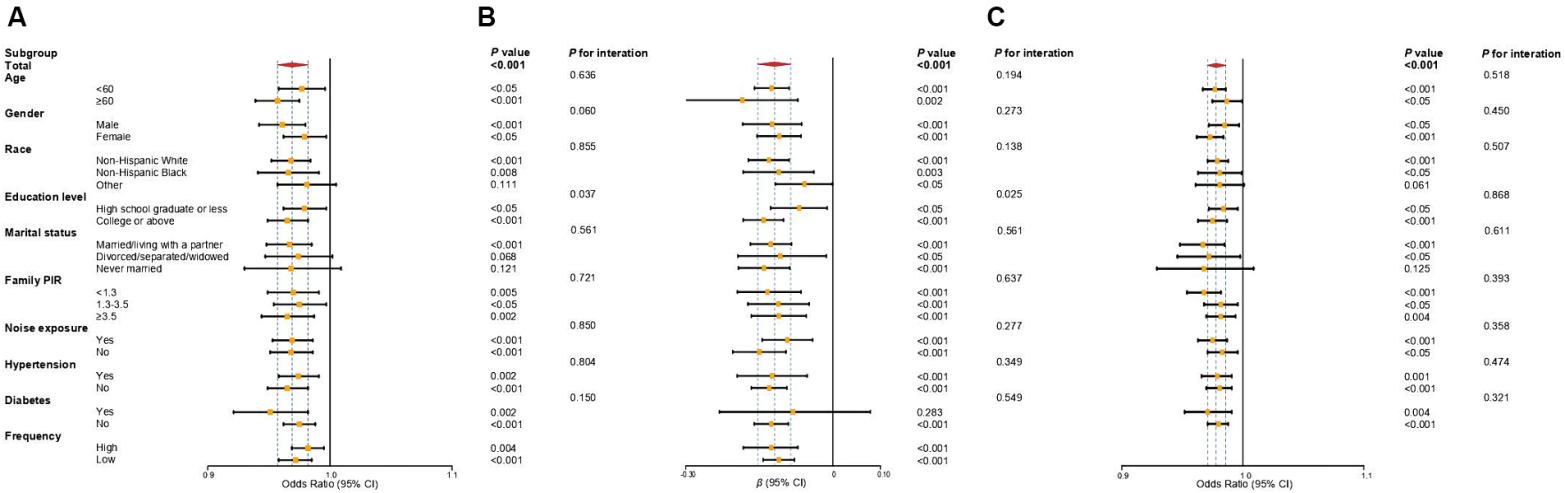
Forest plot of subgroup analysis of the association between OBS and SFHL **(A)**, SF-PTA **(B)**, and tinnitus **(C)**. Odds ratios and regression coefficients (95% confidence intervals) were obtained after individually removing the examined variable from the weighted multivariate logistic regression models and weighted multivariate linear regression models, adjusted for age, gender, race, educational level, marital status, PIR, noise exposure, total energy intake, hypertension, and diabetes. OBS, oxidative balance score; SFHL, speech-frequency hearing loss; SF-PTA, speech-frequency pure-tone average; OR, odds ratios; β, regression coefficient; CI, confidence interval; PIR, income-to-poverty ratio.

Figure 2 and Supplementary Table S4 illustrate the associations between OBS and SFHL, SF-PTA, and tinnitus across different subgroups. The significant negative correlation between OBS and SFHL was observed across all covariate subgroups, except for individuals categorized as “Other” race, those who are divorced/separated/widowed, or never married. This indicates that the protective effect of OBS against SFHL remains consistent across different subgroups of age, gender, racial background (non-Hispanic white or non-Hispanic black), educational level, income level, as well as noise exposure, hypertension, and diabetes status. Concerning SF-PTA, the inverse linear correlation between OBS and SF-PTA was non-significant solely within the subgroup of participants with diabetes. For tinnitus, the negative correlation between OBS and tinnitus was significant across all covariate subgroups, except for those categorized as “Other” race, and individuals who are never married, which is similar to the results observed for OBS and SFHL.

In the interaction analyses of SFHL and SF-PTA, the interaction *p*-values for educational level were 0.037 and 0.025, respectively, indicating that the negative association of OBS with SFHL or SF-PTA was significantly more pronounced in individuals who are college students or above compared to those who are high school graduates or less. Other interactions in the analyses of SFHL and SF-PTA were not statistically significant. For the analyses of tinnitus, all interactions were not statistically significant. These findings of subgroup and interaction analysis suggest the consistency of the negative correlation between OBS and SFHL, SF-PTA, and tinnitus across subgroups.

### 3.5 Sensitivity analysis

Sensitivity analyses were conducted to verify the stability of our results. After dividing OBS into tertiles and quintiles, weighted multivariate logistic regression and weighted multivariate linear regression analyses were performed for SFHL, SF-PTA, and tinnitus, with results presented in Supplementary Tables S5, S6. Within the tertiles of OBS, the results were largely consistent with those observed in the quartile analysis mentioned earlier. In the quintiles of OBS, except for the lack of statistical difference between the second quintile and the lowest quintile, the remaining outcomes were similar to the quartile analysis. Both the tertiles and quintiles of OBS showed statistically significant decreasing trends in SFHL, SF-PTA, and tinnitus (all *p* for trend <0.001). Additionally, the association between OBS and hearing loss as well as tinnitus in imputed data, which indicated a similar trend to our previous findings, was presented in Supplementary Table S7. These findings underscore the robust stability of our results.

### 3.6 Restricted cubic spline regression analysis

**Figure 3:**
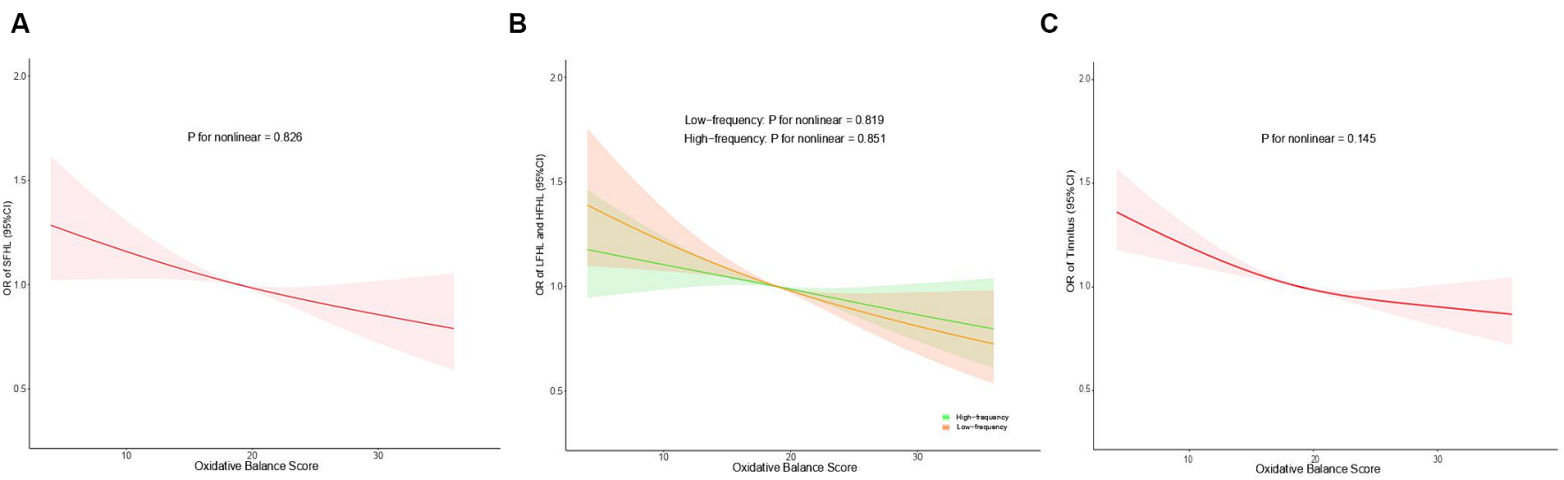
Restricted cubic spline regression analysis of the association between OBS and SFHL **(A)**, LFHL and HFHL **(B)**, and tinnitus **(C)**. Odds ratios (95% confidence intervals) were obtained from the weighted multivariate logistic regression models, adjusted for age, gender, race, educational level, marital status, PIR, noise exposure, total energy intake, hypertension, and diabetes. The solid lines and shaded areas represent the central risk estimates and 95% CIs. OBS, oxidative balance score; SFHL, speech-frequency hearing loss; LFHL, low-frequency hearing loss; HFHL, high-frequency hearing loss; OR, odds ratios; CI, confidence interval; PIR, income-to-poverty ratio.

As illustrated in Figure 3, we employed the RCS models to examine the relationships between OBS and hearing loss (SFHL, LFHL, and HFHL) as well as tinnitus. There was a negative linear association of OBS with hearing loss (SFHL, LFHL, and HFHL) as well as tinnitus. As OBS increases, the OR for hearing loss (SFHL, LFHL, and HFHL) and tinnitus decreases, indicating a diminished risk of suffering from these auditory conditions. In the RCS models for LFHL and HFHL, the OR values for HFHL showed less change with OBS compared to LFHL, suggesting that OBS may have a more pronounced preventive effect on LFHL than on HFHL.

## 4 Discussion

To our knowledge, our study is the first to employ the OBS index to assess the protective effects of the body’s overall antioxidant capacity derived from various dietary and lifestyle factors on hearing loss at speech, low, and high frequencies, along with tinnitus. Our findings demonstrate a negative association between OBS and the risk of hearing loss and tinnitus. This suggests that a higher OBS, indicative of greater antioxidant levels in the body, acts as a protective factor against hearing loss and tinnitus. Furthermore, our analysis reveals that both independent dietary OBS and lifestyle OBS exert protective effects against hearing loss and tinnitus. Additionally, the impact of OBS appears to be more prominent on LFHL compared to HFHL.

In the process of auditory perception, the hair cells situated in the organ of Corti within the cochlea are essential in converting sound energy into neural signals, and they are non-regenerative cells (6). The insufficiency of the antioxidant defenses to clear excessive ROS within cells leads to OS. OS can induce apoptosis and necrosis of hair cells by disrupting intracellular macromolecules and impairing mitochondrial function. These processes are implicated in the development of hearing loss and tinnitus (29–31). While the specific pathophysiological mechanisms underlying various types of hearing loss and tinnitus may vary, OS emerges as a central player (26). Increasing evidence suggests that OS is associated with hearing loss arising from different etiologies, such as age-related, noise-induced, and diabetes-induced hearing loss (24, 48, 49), as well as tinnitus, with our analysis revealing significant negative correlations between OBS and these types of hearing loss and tinnitus. Among them, the inverse linear correlation between OBS and SF-PTA is not significant within the subgroup of participants with diabetes. It is likely due to the high levels of ROS and OS in diabetic patients (50), which reduce the protective effects of OBS. However, the significant negative correlation between OBS and SFHL in diabetic patients indicates that the protective effects of OBS are still meaningful. Our findings indicate that the overall antioxidant capacity derived from dietary and lifestyle factors serves to neutralize ROS and thereby protects the hair cells against oxidative damage (26).

An interesting phenomenon observed is the differential impact of OBS on HFHL and LFHL. In the weighted multivariate logistic regression and RCS regression analyses of HFHL and LFHL, the influence of OBS was found to be more pronounced on LFHL compared to HFHL. Histologically, the hair cells within the cochlea exhibit differential responses to sound frequencies: they are predominantly sensitive to low-frequency sounds at the apex and to high-frequency sounds at the base (51, 52). Based on our findings, it can be inferred that the antioxidant properties of OBS primarily affect the hair cells at the cochlear apex. Common forms of hearing loss, such as age-related, drug-induced, and noise-induced hearing loss, are closely associated with OS (30, 48, 53). In the pathogenesis of these conditions, the susceptibility of hair cells to OS follows a base-to-apex pattern (30, 48, 53–55), resulting primarily in damage and apoptosis in the basal hair cells. These damages may be early and severe, making it challenging for the antioxidant capacity of OBS to provide apparent protection. In contrast, apical hair cells, which receive low-frequency sounds, are less affected by OS, thereby demonstrating a more pronounced protective effect of OBS. Another possible explanation is that apical hair cells, being farthest from the blood supply to the cochlea, are more susceptible to the effects of microcirculation in the stria vascularis. The various components of OBS, such as folic acid, fat, and physical activity, may not only possess antioxidant properties but also potentially protect low-frequency hearing by improving microcirculation, which consequently demonstrates a protective effect on low-frequency hearing (56–58). Additionally, the weaker protective effects of OBS on HFHL may be attributed to the higher biological variability associated with high-frequency thresholds compared to those of low frequencies. Further research is required to elucidate the differential protective effects of OBS on the apical and basal hair cells of the cochlea.

Based on the important involvement of OS and antioxidants in hearing loss and tinnitus, an expanding body of research supports the effectiveness of various dietary or lifestyle factors with antioxidant properties in protecting against hearing loss and tinnitus (24, 25). These dietary factors, such as carotene, vitamins B12, C, and E, and magnesium, along with lifestyle factors such as smoking, alcohol consumption, and physical activity, are all components of the OBS. Most studies have primarily examined the relationship between individual or specific combinations of these antioxidant factors and hearing loss or tinnitus, rather than evaluating total antioxidant capacity comprehensively (24, 25). A recent study investigated the association between dietary total antioxidant capacity (dTAC) and hearing loss (59). However, in the overall population, there was no significant correlation observed between elevated dTAC levels and a decreased risk of hearing loss. Possible explanations for this observation include a relatively small proportion of elderly participants, insufficient data on noise exposure, and non-representative study populations. Additionally, a notable limitation is the absence of an assessment of lifestyle antioxidant capacity. The effective combination of dietary and lifestyle factors has been demonstrated in various age-related or chronic diseases, including tinnitus (60–64). Therefore, it is meaningful to investigate the association of the combined antioxidant capacity of dietary and lifestyle factors with hearing loss and tinnitus. The OBS was formulated to assess body’s overall oxidative balance using 16 dietary and 4 lifestyle components, comprising 5 pro-oxidants and 15 antioxidants (34). Our study illustrates an inverse correlation between OBS and hearing loss as well as tinnitus, providing support for tailored dietary and lifestyle interventions aimed at mitigating these auditory conditions. The universal exposure of dietary and lifestyle factors suggests that tailored dietary and lifestyle interventions could significantly impact hearing loss and tinnitus in the population.

Our study possesses several significant strengths. Our study is the first to use the OBS index to assess the protective effects of the body’s overall antioxidant capacity derived from various dietary and lifestyle factors on hearing loss at speech, low, and high frequencies, as well as tinnitus. By concurrently investigating the associations between OBS and hearing loss (SFHL, LFHL, and HFHL), as well as the associations between OBS and PTA (SF-PTA, LF-PTA, and HF-PTA), we enhanced the robustness of our findings. Furthermore, our study is based on NHANES data spanning from 1999 to 2018, encompassing individuals aged 20 and above. This ensures the representativeness of the study population and the generalizability of the obtained results. Additionally, we included several important covariates based on prior research, reducing the impact of confounding variables. Lastly, we conducted subgroup and sensitivity analyses, which affirmed the consistency and stability of our findings.

Some limitations should be noted. Firstly, our study did not categorize hearing loss and tinnitus by cause, thereby constraining our comprehension of the association between different types of hearing loss as well as tinnitus and OBS. Secondly, its cross-sectional design prevents the establishment of causality between OBS and hearing loss as well as tinnitus, which needs future longitudinal research. Moreover, our study did not account for the contribution of antioxidant intake from dietary supplements. Additionally, potential sources of bias inherent in using self-reported diet and lifestyle data from NHANES cannot be ignored. Finally, potential alterations in dietary and lifestyle patterns over time were not considered, which may potentially influence OBS and its association with hearing loss as well as tinnitus.

## 5 Conclusion

This comprehensive cross-sectional study has demonstrated a significant negative association between OBS and hearing loss (SFHL, LFHL, and HFHL) as well as tinnitus. The protective effect of OBS against SFHL and tinnitus remains consistent across different groups of age, gender, racial background (non-Hispanic white or non-Hispanic black), educational level, income level, as well as noise exposure, hypertension, and diabetes status. These findings underscore the potential of antioxidant diets and lifestyles based on OBS in mitigating the risk of hearing loss and tinnitus. Therefore, combined antioxidant dietary and lifestyle interventions hold promise as potential strategies for reducing the prevalence of hearing loss and tinnitus. Future prospective and experimental studies are necessary to validate the causal relationship and elucidate the precise mechanisms of the association between OBS and hearing loss as well as tinnitus.

## Data availability statement

Publicly available datasets were analyzed in this study. This data can be found here: National Health and Nutrition Examination Surveys database (https://www.cdc.gov/nchs/nhanes/index.htm).

## Ethics statement

The studies involving humans were approved by Research Ethics Reviewer Board of the National Center for Health Statistics (NCHS). The studies were conducted in accordance with the local legislation and institutional requirements. The participants provided their written informed consent to participate in this study.

## Author contributions

HL: Data curation, Methodology, Writing – original draft. JY: Methodology, Writing – review & editing. HY: Conceptualization, Writing – review & editing.

## References

[1] Chadha S Kamenov K Cieza A. The world report on hearing, 2021. Bull World Health Organ. (2021) 99:242-A. doi: 10.2471/BLT.21.285643 33953438 PMC8085630

[2] Disease GBD Injury I Prevalence C. Global, regional, and national incidence, prevalence, and years lived with disability for 310 diseases and injuries, 1990–2015: a systematic analysis for the global burden of Disease study 2015. Lancet. (2016) 388:1545–602. doi: 10.1016/S0140-6736(16)31678-6 27733282 PMC5055577

[3] Rutherford BR Brewster K Golub JS Kim AH Roose SP. Sensation and psychiatry: linking age-related hearing loss to late-life depression and cognitive decline. Am J Psychiatry. (2018) 175:215–24. doi: 10.1176/appi.ajp.2017.17040423, PMID: 29202654 PMC5849471

[4] Chakrabarty S Mudar R Chen Y Husain FT. Contribution of tinnitus and hearing loss to depression: NHANES population study. Ear Hear. (2024) 45:775–86. doi: 10.1097/AUD.0000000000001467, PMID: 38291574

[5] Chen X Hu K Song H Yin L Kaijser M Gurholt TP. Depression, anxiety and brain volume after hearing loss and tinnitus: cohort study in the UK biobank. BJPsych Open. (2024) 10:e37. doi: 10.1192/bjo.2023.634, PMID: 38297917 PMC10897703

[6] Cunningham LL Tucci DL. Hearing loss in adults. N Engl J Med. (2017) 377:2465–73. doi: 10.1056/NEJMra1616601 29262274 PMC6457651

[7] Haider HF Bojic T Ribeiro SF Paco J Hall DA Szczepek AJ. Pathophysiology of subjective tinnitus: triggers and maintenance. Front Neurosci. (2018) 12:866. doi: 10.3389/fnins.2018.00866, PMID: 30538616 PMC6277522

[8] Langguth B Kreuzer PM Kleinjung T De Ridder D. Tinnitus: causes and clinical management. Lancet Neurol. (2013) 12:920–30. doi: 10.1016/S1474-4422(13)70160-1 23948178

[9] Jarach CM Lugo A Scala M van den Brandt PA Cederroth CR Odone A. Global Prevalence and incidence of tinnitus: a systematic review and Meta-analysis. JAMA Neurol. (2022) 79:888–900. doi: 10.1001/jamaneurol.2022.2189, PMID: 35939312 PMC9361184

[10] Baguley D McFerran D Hall D. Tinnitus. Lancet. (2013) 382:1600–7. doi: 10.1016/S0140-6736(13)60142-7 23827090

[11] Cruickshanks KJ Klein R Klein BE Wiley TL Nondahl DM Tweed TS. Cigarette smoking and hearing loss: the epidemiology of hearing loss study. JAMA. (1998) 279:1715–9. doi: 10.1001/jama.279.21.1715 9624024

[12] Veile A Zimmermann H Lorenz E Becher H. Is smoking a risk factor for tinnitus? A systematic review, meta-analysis and estimation of the population attributable risk in Germany. BMJ Open. (2018) 8:e016589. doi: 10.1136/bmjopen-2017-016589, PMID: 29472253 PMC5855477

[13] Qian P Zhao Z Liu S Xin J Liu Y Hao Y. Alcohol as a risk factor for hearing loss: a systematic review and meta-analysis. PLoS One. (2023) 18:e0280641. doi: 10.1371/journal.pone.0280641, PMID: 36662896 PMC9858841

[14] Kawakami R Sawada SS Kato K Gando Y Momma H Oike H. A prospective cohort study of muscular and performance fitness and risk of hearing loss: the Niigata wellness study. Am J Med. (2021) 134:235–242.e4. doi: 10.1016/j.amjmed.2020.06.021, PMID: 32687815

[15] Chen S Yang X Jiang Y Wu F Li Y Qiu J. Associations between physical activity, tinnitus, and tinnitus severity. Ear Hear. (2023) 44:619–26. doi: 10.1097/AUD.0000000000001306, PMID: 36404413

[16] Kim TS Chung JW. Associations of dietary riboflavin, niacin, and retinol with age-related hearing loss: an analysis of Korean National Health and nutrition examination survey data. Nutrients. (2019) 11:896. doi: 10.3390/nu11040896 31010085 PMC6520829

[17] Kang JW Choi HS Kim K Choi JY. Dietary vitamin intake correlates with hearing thresholds in the older population: the Korean National Health and nutrition examination survey. Am J Clin Nutr. (2014) 99:1407–13. doi: 10.3945/ajcn.113.072793, PMID: 24646817

[18] Rodrigo L Campos-Asensio C Rodriguez MA Crespo I Olmedillas H. Role of nutrition in the development and prevention of age-related hearing loss: a scoping review. J Formos Med Assoc. (2021) 120:107–20. doi: 10.1016/j.jfma.2020.05.011, PMID: 32473863

[19] Jarach CM Lugo A Garavello W van den Brandt PA Odone A Cederroth CR. The role of diet in tinnitus onset: a hospital-based case-control study from Italy. Nutrients. (2023) 15:621. doi: 10.3390/nu15030621, PMID: 36771329 PMC9920666

[20] Marcrum SC Engelke M Goedhart H Langguth B Schlee W Vesala M. The influence of diet on tinnitus severity: results of a large-scale, online survey. Nutrients. (2022) 14:5356. doi: 10.3390/nu14245356, PMID: 36558515 PMC9784733

[21] Jin Y Tanaka T Reed NS Tucker KL Ferrucci L Talegawkar SA. Associations between dietary indices and hearing status among middle-older aged adults- results from the Baltimore longitudinal study of aging. Am J Clin Nutr. (2024) 119:1338–45. doi: 10.1016/j.ajcnut.2024.03.001, PMID: 38447686 PMC11130650

[22] Yévenes-Briones H Caballero FF Struijk EA Machado-Fragua MD Ortolá R Rodríguez-Artalejo F. Diet quality and the risk of impaired speech reception threshold in noise: the UK biobank cohort. Ear Hear. (2022) 43:361–9. doi: 10.1097/AUD.0000000000001108, PMID: 34320526

[23] Spankovich C Bishop C Johnson MF Elkins A Su D Lobarinas E. Relationship between dietary quality, tinnitus and hearing level: data from the national health and nutrition examination survey, 1999–2002. Int J Audiol. (2017) 56:716–22. doi: 10.1080/14992027.2017.1331049, PMID: 28553744

[24] Tang D Tran Y Dawes P Gopinath B. A narrative review of lifestyle risk factors and the role of oxidative stress in age-related hearing loss. Antioxidants (Basel). (2023) 12:878. doi: 10.3390/antiox12040878 37107253 PMC10135296

[25] Chen HL Tan CT Wu CC Liu TC. Effects of diet and lifestyle on audio-vestibular dysfunction in the elderly: a literature review. Nutrients. (2022) 14:4720. doi: 10.3390/nu14224720, PMID: 36432406 PMC9698578

[26] Kishimoto-Urata M Urata S Fujimoto C Yamasoba T. Role of oxidative stress and antioxidants in acquired inner ear disorders. Antioxidants (Basel). (2022) 11:1469. doi: 10.3390/antiox11081469 36009187 PMC9405327

[27] Neri S Signorelli S Pulvirenti D Mauceri B Cilio D Bordonaro F. Oxidative stress, nitric oxide, endothelial dysfunction and tinnitus. Free Radic Res. (2006) 40:615–8. doi: 10.1080/10715760600623825, PMID: 16753839

[28] Jones DP. Radical-free biology of oxidative stress. Am J Physiol Cell Physiol. (2008) 295:C849–68. doi: 10.1152/ajpcell.00283.2008, PMID: 18684987 PMC2575825

[29] Tan WJT Song L. Role of mitochondrial dysfunction and oxidative stress in sensorineural hearing loss. Hear Res. (2023) 434:108783. doi: 10.1016/j.heares.2023.108783, PMID: 37167889

[30] Benkafadar N François F Affortit C Casas F Ceccato JC Menardo J. ROS-induced activation of DNA damage responses drives senescence-like state in Postmitotic Cochlear cells: implication for hearing preservation. Mol Neurobiol. (2019) 56:5950–69. doi: 10.1007/s12035-019-1493-6, PMID: 30693443 PMC6614136

[31] Li P Li S Wang L Li H Wang Y Liu H. Mitochondrial dysfunction in hearing loss: oxidative stress, autophagy and NLRP3 inflammasome. Front Cell Dev Biol. (2023) 11:1119773. doi: 10.3389/fcell.2023.1119773 36891515 PMC9986271

[32] He Y Zheng Z Liu C Li W Zhao L Nie G. Inhibiting DNA methylation alleviates cisplatin-induced hearing loss by decreasing oxidative stress-induced mitochondria-dependent apoptosis via the LRP1-PI3K/AKT pathway. Acta Pharm Sin B. (2022) 12:1305–21. doi: 10.1016/j.apsb.2021.11.002, PMID: 35530135 PMC9069410

[33] Petridou AI Zagora ET Petridis P Korres GS Gazouli M Xenelis I. The effect of antioxidant supplementation in patients with tinnitus and Normal hearing or hearing loss: a randomized, double-blind, placebo controlled trial. Nutrients. (2019) 11:3037. doi: 10.3390/nu11123037, PMID: 31842394 PMC6950042

[34] Hernández-Ruiz Á García-Villanova B Guerra-Hernández EJ Carrión-García CJ Amiano P Sánchez MJ. Oxidative balance scores (OBSs) integrating nutrient, food and lifestyle dimensions: development of the NutrientL-OBS and FoodL-OBS. Antioxidants (Basel). (2022) 11:300. doi: 10.3390/antiox11020300 35204183 PMC8868253

[35] Liu X Liu X Wang Y Zeng B Zhu B Dai F. Association between depression and oxidative balance score: National Health and nutrition examination survey (NHANES) 2005–2018. J Affect Disord. (2023) 337:57–65. doi: 10.1016/j.jad.2023.05.071, PMID: 37244542

[36] Chen X Wang C Dong Z Luo H Ye C Li L. Interplay of sleep patterns and oxidative balance score on total cardiovascular disease risk: insights from the National Health and nutrition examination survey 2005–2018. J Glob Health. (2023) 13:04170. doi: 10.7189/jogh.14.04170, PMID: 38085249 PMC10715456

[37] Ke R He Y Chen C. Association between oxidative balance score and kidney stone in United States adults: analysis from NHANES 2007–2018. Front Physiol. (2023) 14:1275750. doi: 10.3389/fphys.2023.1275750, PMID: 38028789 PMC10654971

[38] Liu X Wang Y Liu X Zeng B Zhu B Zhang Y. Higher oxidative balance scores are associated with lower nonalcoholic fatty liver disease and not with fibrosis in US adults. Nutr Metab Cardiovasc Dis. (2023) 33:2488–96. doi: 10.1016/j.numecd.2023.08.004 37798234

[39] Lei X Xu Z Chen W. Association of oxidative balance score with sleep quality: NHANES 2007–2014. J Affect Disord. (2023) 339:435–42. doi: 10.1016/j.jad.2023.07.040, PMID: 37442450

[40] World Health Organization. World report on hearing. Geneva: World Health Organization (2021).

[41] Helzner EP Cauley JA Pratt SR Wisniewski SR Zmuda JM Talbott EO. Race and sex differences in age-related hearing loss: the health, aging and body composition study. J Am Geriatr Soc. (2005) 53:2119–27. doi: 10.1111/j.1532-5415.2005.00525.x, PMID: 16398896

[42] Basner M Babisch W Davis A Brink M Clark C Janssen S. Auditory and non-auditory effects of noise on health. Lancet. (2014) 383:1325–32. doi: 10.1016/S0140-6736(13)61613-X, PMID: 24183105 PMC3988259

[43] Shore SE Roberts LE Langguth B. Maladaptive plasticity in tinnitus--triggers, mechanisms and treatment. Nat Rev Neurol. (2016) 12:150–60. doi: 10.1038/nrneurol.2016.12, PMID: 26868680 PMC4895692

[44] Lin BM Curhan SG Wang M Eavey R Stankovic KM Curhan GC. Hypertension, diuretic use, and risk of hearing loss. Am J Med. (2016) 129:416–22. doi: 10.1016/j.amjmed.2015.11.014, PMID: 26656761 PMC4792671

[45] Figueiredo RR Azevedo AA Penido NO. Positive association between tinnitus and arterial hypertension. Front Neurol. (2016) 7:171. doi: 10.3389/fneur.2016.00171, PMID: 27761128 PMC5050200

[46] Gupta S Eavey RD Wang M Curhan SG Curhan GC. Type 2 diabetes and the risk of incident hearing loss. Diabetologia. (2019) 62:281–5. doi: 10.1007/s00125-018-4766-0 30402776 PMC6494103

[47] Gibrin PC Melo JJ Marchiori LL. Prevalence of tinnitus complaints and probable association with hearing loss, diabetes mellitus and hypertension in elderly. Codas. (2013) 25:176–80. doi: 10.1590/S2317-17822013000200014, PMID: 24408248

[48] Fetoni AR Paciello F Rolesi R Paludetti G Troiani D. Targeting dysregulation of redox homeostasis in noise-induced hearing loss: oxidative stress and ROS signaling. Free Radic Biol Med. (2019) 135:46–59. doi: 10.1016/j.freeradbiomed.2019.02.022, PMID: 30802489

[49] Aladag I Eyibilen A Guven M Atis O Erkorkmaz U. Role of oxidative stress in hearing impairment in patients with type two diabetes mellitus. J Laryngol Otol. (2009) 123:957–63. doi: 10.1017/S0022215109004502, PMID: 19203398

[50] Zhang P Li T Wu X Nice EC Huang C Zhang Y. Oxidative stress and diabetes: antioxidative strategies. Front Med. (2020) 14:583–600. doi: 10.1007/s11684-019-0729-1 32248333

[51] Defourny J Lallemend F Malgrange B. Structure and development of cochlear afferent innervation in mammals. Am J Physiol Cell Physiol. (2011) 301:C750–61. doi: 10.1152/ajpcell.00516.2010, PMID: 21753183

[52] Li H Helpard L Ekeroot J Rohani SA Zhu N Rask-Andersen H. Three-dimensional tonotopic mapping of the human cochlea based on synchrotron radiation phase-contrast imaging. Sci Rep. (2021) 11:4437. doi: 10.1038/s41598-021-83225-w, PMID: 33627724 PMC7904830

[53] Rybak LP Ramkumar V. Ototoxicity. Kidney Int. (2007) 72:931–5. doi: 10.1038/sj.ki.5002434 17653135

[54] Cao Z Yang Q Yin H Qi Q Li H Sun G. Peroxynitrite induces apoptosis of mouse cochlear hair cells via a caspase-independent pathway in vitro. Apoptosis. (2017) 22:1419–30. doi: 10.1007/s10495-017-1417-8 28900799

[55] Yang Y Chen X Tian C Fan B An X Liu Z. Gene expression analysis of oxidative stress-related genes in the apical, middle, and basal turns of the cochlea. Gene Expr Patterns. (2024) 51:119356. doi: 10.1016/j.gep.2024.119356, PMID: 38432189

[56] Durga J Verhoef P Anteunis LJ Schouten E Kok FJ. Effects of folic acid supplementation on hearing in older adults: a randomized, controlled trial. Ann Intern Med. (2007) 146:1–9. doi: 10.7326/0003-4819-146-1-200701020-00003, PMID: 17200216

[57] Hwang JH Hsu CJ Yu WH Liu TC Yang WS. Diet-induced obesity exacerbates auditory degeneration via hypoxia, inflammation, and apoptosis signaling pathways in CD/1 mice. PLoS One. (2013) 8:e60730. doi: 10.1371/journal.pone.0060730, PMID: 23637762 PMC3637206

[58] Han C Ding D Lopez MC Manohar S Zhang Y Kim MJ. Effects of long-term exercise on age-related hearing loss in mice. J Neurosci. (2016) 36:11308–19. doi: 10.1523/JNEUROSCI.2493-16.2016, PMID: 27807171 PMC5148246

[59] Ghosn B Azadbakht L Esmaeilpour MRM Esmaillzadeh A. The association between dietary total antioxidant capacity and hearing loss: results from the Tehran employees cohort study. BMC Public Health. (2024) 24:818. doi: 10.1186/s12889-024-18108-6, PMID: 38491357 PMC10941599

[60] the EVIDENT Group García-Ortiz L Recio-Rodríguez JI Martín-Cantera C Cabrejas-Sánchez A Gómez-Arranz A. Physical exercise, fitness and dietary pattern and their relationship with circadian blood pressure pattern, augmentation index and endothelial dysfunction biological markers: EVIDENT study protocol. BMC Public Health. (2010) 10:233. doi: 10.1186/1471-2458-10-233, PMID: 20459634 PMC2881095

[61] Denison HJ Cooper C Sayer AA Robinson SM. Prevention and optimal management of sarcopenia: a review of combined exercise and nutrition interventions to improve muscle outcomes in older people. Clin Interv Aging. (2015) 10:859–69. doi: 10.2147/CIA.S55842, PMID: 25999704 PMC4435046

[62] Gawel E Hall B Siatkowski S Grabowska A Zwierzchowska A. The combined effects of high-intensity interval exercise training and dietary supplementation on reduction of body fat in adults with overweight and obesity: a systematic review. Nutrients. (2024) 16:355. doi: 10.3390/nu16030355, PMID: 38337640 PMC10857230

[63] Ozbey-Yucel U Aydogan Z Tokgoz-Yilmaz S Ucar A Ocak E Beton S. The effects of diet and physical activity induced weight loss on the severity of tinnitus and quality of life: a randomized controlled trial. Clin Nutr ESPEN. (2021) 44:159–65. doi: 10.1016/j.clnesp.2021.05.010, PMID: 34330461

[64] Ozbey-Yucel U Ucar A Aydogan Z Tokgoz-Yilmaz S Beton S. The effects of dietary and physical activity interventions on tinnitus symptoms: an RCT. Auris Nasus Larynx. (2023) 50:40–7. doi: 10.1016/j.anl.2022.04.013, PMID: 35568580

