# Supplementary material for: Association of oxidative balance score with hearing loss and tinnitus: NHANES 1999–2018
Source: Front Nutr. 2024 Jun 19;11:1421605. doi: 10.3389/fnut.2024.1421605 (PMC11220283; doi:10.3389/fnut.2024.1421605)
Supplement: Supplementary file 1 [file Table_1.DOCX]

Supplementary Table S1. Oxidative balance score assignment scheme in individuals with or without hearing loss

| OBS components | Property | Male | | | Female | | |
| --- | --- | --- | --- | --- | --- | --- | --- |
|  |  | 0 | 1 | 2 | 0 | 1 | 2 |
| **Dietary OBS components** | | | | | | | |
| Dietary fiber (g/d) | A | <12.60 | 12.60-21.10 | ≥21.10 | <10.30 | 10.30-17.10 | ≥17.10 |
| Carotene (RE/d) | A | <91.67 | 91.67-326.50 | ≥326.50 | <88.33 | 88.33-378.50 | ≥378.50 |
| Riboflavin (mg/d) | A | <1.82 | 1.82-2.75 | ≥2.75 | <1.36 | 1.36-2.09 | ≥2.09 |
| Niacin (mg/d) | A | <21.91 | 21.91-33.06 | ≥33.06 | <15.30 | 15.30-23.16 | ≥23.16 |
| Vitamin B_6_ (mg/d) | A | <1.63 | 1.63-2.57 | ≥2.57 | <1.17 | 1.17-1.88 | ≥1.88 |
| Total folate (mcg/d) | A | <319.00 | 319.00-506.00 | ≥506.00 | <241.00 | 241.00-390.00 | ≥390.00 |
| Vitamin B_12_ (mcg/d) | A | <3.43 | 3.43-6.39 | ≥6.39 | <2.29 | 2.29-4.45 | ≥4.45 |
| Vitamin C (mg/d) | A | <31.20 | 31.20-95.00 | ≥95.00 | <29.70 | 29.70-85.50 | ≥85.50 |
| Vitamin E (ATE) (mg/d) | A | <5.97 | 5.97-10.32 | ≥10.32 | <4.84 | 4.84-8.22 | ≥8.22 |
| Calcium (mg/d) | A | <697.00 | 697.00-1175.00 | ≥1175.00 | <560.00 | 560.00-941.00 | ≥941.00 |
| Magnesium (mg/d) | A | <257.00 | 257.00-376.36 | ≥376.36 | <200.00 | 200.00-293.00 | ≥293.00 |
| Zinc (mg/d) | A | <9.70 | 9.70-15.05 | ≥15.05 | <6.94 | 6.94-10.79 | ≥10.79 |
| Copper (mg/d) | A | <1.04 | 1.04-1.56 | ≥1.56 | <0.84 | 0.84-1.25 | ≥1.25 |
| Selenium (mcg/d) | A | <99.50 | 99.50-146.20 | ≥146.20 | <70.00 | 70.00-106.20 | ≥106.20 |
| Total fat (g/d) | P | ≥70.47 | 70.47-110.77 | <110.77 | ≥52.28 | 52.28-80.54 | <80.54 |
| Iron (mg/d) | P | ≥12.43 | 12.43-18.98 | <18.98 | ≥9.28 | 9.28-14.20 | <14.20 |
| **Lifestyle OBS components** | | | | |  |  |  |
| Physical activity (MET-minute/week) | A | <1071.00 | 1071.00-3720.00 | ≥3720.00 | <760.00 | 760.00-2480.00 | ≥2480.00 |
| Alcohol (g/d) | P | ≥30 | 0-30 | None | ≥15 | 0-15 | None |
| Body mass index (kg/m^2^) | P | ≥25.70 | 25.70-29.98 | <29.98 | ≥24.84 | 24.84-30.80 | <30.80 |
| Cotinine (ng/mL) | P | ≥0.027 | 0.027-2.040 | <2.040 | ≥0.016 | 0.016-0.109 | <0.109 |

Abbreviations: OBS, oxidative balance score; A, antioxidant; P, pro-oxidant; RE, retinol equivalent; ATE, alpha-tocopherol equivalent; MET, metabolic equivalent.

Supplementary Table S2. Oxidative balance score assignment scheme in individuals with or without tinnitus

| OBS components | Property | Male | | | Female | | |
| --- | --- | --- | --- | --- | --- | --- | --- |
|  |  | 0 | 1 | 2 | 0 | 1 | 2 |
| **Dietary OBS components** | | | | | | | |
| Dietary fiber (g/d) | A | <12.40 | 12.40-20.50 | ≥20.50 | <10.00 | 10.00-16.50 | ≥16.50 |
| Carotene (RE/d) | A | <92.50 | 92.50-326.28 | ≥326.28 | <88.50 | 88.50-382.17 | ≥382.17 |
| Riboflavin (mg/d) | A | <1.79 | 1.79-2.73 | ≥2.73 | <1.34 | 1.34-2.06 | ≥2.06 |
| Niacin (mg/d) | A | <21.31 | 21.31-32.04 | ≥32.04 | <14.69 | 14.69-22.60 | ≥22.60 |
| Vitamin B_6_ (mg/d) | A | <1.59 | 1.59-2.51 | ≥2.51 | <1.13 | 1.13-1.82 | ≥1.82 |
| Total folate (mcg/d) | A | <316.00 | 316.00-501.00 | ≥501.00 | <239.00 | 239.00-385.00 | ≥385.00 |
| Vitamin B_12_ (mcg/d) | A | <3.36 | 3.36-6.26 | ≥6.26 | <2.25 | 2.25-4.38 | ≥4.38 |
| Vitamin C (mg/d) | A | <33.30 | 33.30-98.73 | ≥98.73 | <30.40 | 30.40-87.70 | ≥87.70 |
| Vitamin E (ATE) (mg/d) | A | <5.69 | 5.69-9.88 | ≥9.88 | <4.54 | 4.54-7.77 | ≥7.77 |
| Calcium (mg/d) | A | <665.00 | 665.00-1142.46 | ≥1142.46 | <528.00 | 528.00-905.00 | ≥905.00 |
| Magnesium (mg/d) | A | <250.00 | 250.00-369.00 | ≥369.00 | <191.00 | 191.00-284.52 | ≥284.52 |
| Zinc (mg/d) | A | <9.58 | 9.58-15.03 | ≥15.03 | <6.80 | 6.80-10.62 | ≥10.62 |
| Copper (mg/d) | A | <1.04 | 1.04-1.56 | ≥1.56 | <0.82 | 0.82-1.23 | ≥1.23 |
| Selenium (mcg/d) | A | <95.40 | 95.40-144.10 | ≥144.10 | <68.20 | 68.20-103.30 | ≥103.30 |
| Total fat (g/d) | P | ≥69.38 | 69.38-108.81 | <108.81 | ≥50.61 | 50.61-78.52 | <78.52 |
| Iron (mg/d) | P | ≥12.40 | 12.40-19.05 | <19.05 | ≥9.31 | 9.31-14.04 | <14.04 |
| **Lifestyle OBS components** | | | | |  |  |  |
| Physical activity (MET-minute/week) | A | <892.50 | 892.50-2940.00 | ≥2940.00 | <620.00 | 620.00-1974.00 | ≥1974.00 |
| Alcohol (g/d) | P | ≥30 | 0-30 | None | ≥15 | 0-15 | None |
| Body mass index (kg/m^2^) | P | ≥25.60 | 25.60-29.72 | <29.72 | ≥24.65 | 24.65-30.41 | <30.41 |
| Cotinine (ng/mL) | P | ≥0.035 | 0.035-2.460 | <2.460 | ≥0.019 | 0.019-0.120 | <0.120 |

Abbreviations: OBS, oxidative balance score; A, antioxidant; P, pro-oxidant; RE, retinol equivalent; ATE, alpha-tocopherol equivalent; MET, metabolic equivalent.

Supplementary Table S3. Association between OBS and LFHL as well as HFHL.

|  | **Model 1** | | **Model 2** | | **Model 3** | |
| --- | --- | --- | --- | --- | --- | --- |
|  | **OR/β (95%CI)** | **P value** | **OR/β (95%CI)** | **P value** | **OR/β (95%CI)** | **P value** |
| **LFHL** | | | | | | |
| OBS (continuous) | 0.969 (0.959, 0.980) | ＜0.001 | 0.971 (0.957, 0.985) | ＜0.001 | 0.972 (0.958, 0.985) | ＜0.001 |
| OBS (quartile) | | | | | | |
| Q1 | Reference |  | Reference |  | Reference |  |
| Q2 | 0.691 (0.559, 0.853) | ＜0.001 | 0.703 (0.562, 0.880) | 0.002 | 0.707 (0.565, 0.885) | 0.003 |
| Q3 | 0.600 (0.483, 0.744) | ＜0.001 | 0.614 (0.479, 0.787) | ＜0.001 | 0.618 (0.481, 0.794) | ＜0.001 |
| Q4 | 0.539 (0.437, 0.665) | ＜0.001 | 0.563 (0.432, 0.735) | ＜0.001 | 0.573 (0.440, 0.745) | ＜0.001 |
| p for trend | ＜0.001 |  | ＜0.001 |  | ＜0.001 |  |
|  | | | | | | |
| **LF-PTA** | | | | | | |
| OBS (continuous) | -0.135 (-0.164, -0.105) | ＜0.001 | -0.119 (-0.152, -0.085) | ＜0.001 | -0.114 (-0.147, -0.081) | ＜0.001 |
| OBS (quartile) | | | | | | |
| Q1 | Reference |  | Reference |  | Reference |  |
| Q2 | -0.914 (-1.569, -0.259) | 0.007 | -0.699 (-1.364, -0.034) | ＜0.05 | -0.650 (-1.324, 0.025) | 0.059 |
| Q3 | -1.822 (-2.427, -1.217) | ＜0.001 | -1.551 (-2.253, -0.849) | ＜0.001 | -1.494 (-2.199, -0.789) | ＜0.001 |
| Q4 | -2.579 (-3.212, -1.946) | ＜0.001 | -2.199 (-2.939, -1.458) | ＜0.001 | -2.103 (-2.841, -1.366) | ＜0.001 |
| p for trend | ＜0.001 |  | ＜0.001 |  | ＜0.001 |  |
|  | | | | | | |
| **HFHL** | | | | | | |
| OBS (continuous) | 0.979 (0.969, 0.990) | ＜0.001 | 0.978 (0.964, 0.991) | 0.002 | 0.980 (0.966, 0.993) | 0.004 |
| OBS (quartile) | | | | | | |
| Q1 | Reference |  | Reference |  | Reference |  |
| Q2 | 0.881 (0.727, 1.069) | 0.196 | 0.890 (0.726, 1.090) | 0.257 | 0.906 (0.736, 1.115) | 0.348 |
| Q3 | 0.717 (0.595, 0.864) | ＜0.001 | 0.718 (0.586, 0.880) | 0.002 | 0.734 (0.597, 0.903) | 0.004 |
| Q4 | 0.724 (0.586, 0.895) | 0.003 | 0.735 (0.566, 0.953) | ＜0.05 | 0.762 (0.588, 0.988) | ＜0.05 |
| p for trend | ＜0.001 |  | 0.006 |  | ＜0.05 |  |
|  | | | | | | |
| **HF-PTA** | | | | | | |
| OBS (continuous) | -0.175 (-0.227, -0.124) | ＜0.001 | -0.136 (-0.192, -0.080) | ＜0.001 | -0.130 (-0.185, -0.074) | ＜0.001 |
| OBS (quartile) | | | | | | |
| Q1 | Reference |  | Reference |  | Reference |  |
| Q2 | -1.076 (-2.167, 0.014) | 0.053 | -0.651 (-1.723, 0.421) | 0.231 | -0.582 (-1.674, 0.510) | 0.293 |
| Q3 | -2.657 (-3.587, -1.728) | ＜0.001 | -2.047 (-3.014, -1.079) | ＜0.001 | -1.967 (-2.950, -0.984) | ＜0.001 |
| Q4 | -3.075 (-4.155, -1.996) | ＜0.001 | -2.123 (-3.288, -0.957) | ＜0.001 | -1.994 (-3.169, -0.818) | ＜0.001 |
| p for trend | ＜0.001 |  | ＜0.001 |  | ＜0.001 |  |

Model 1: Adjusted for age, gender, and race.

Model 2: Combination of model 1 and educational level, marital status, PIR, noise exposure, and total energy intake.

Model 3: Combination of model 2 and hypertension, and diabetes.

Abbreviations: OBS, oxidative balance score; Q, quartile; LFHL, low-frequency hearing loss; LF-PTA, low-frequency pure-tone average; HFHL, high-frequency hearing loss; HF-PTA, high-frequency pure-tone average; OR: odds ratios; β: regression coefficient; CI: confidence interval; PIR, income-to-poverty ratio

Supplementary Table S4. Subgroup analysis of the association between OBS and hearing loss as well as tinnitus.

| **Subgroup** | **Positive Proportion / Mean** | **OBS [OR/β (95%CI) ]** | ***P* value** | ***P* for interaction** |
| --- | --- | --- | --- | --- |
| **SFHL** | | | | |
| Total | 3455/13715 | 0.969 (0.957, 0.982) | <0.001 |  |
| Age |  |  |  | 0.636 |
| <60 | 882/9422 | 0.977 (0.958, 0.996) | <0.05 |  |
| ≥60 | 2573/4293 | 0.957 (0.939, 0.975) | <0.001 |  |
| Gender |  |  |  | 0.060 |
| Male | 2097/6730 | 0.961 (0.942, 0.980) | <0.001 |  |
| Female | 1358/6985 | 0.979 (0.962, 0.997) | <0.05 |  |
| Race |  |  |  | 0.855 |
| Non-Hispanic White | 1996/6080 | 0.968 (0.952, 0.984) | <0.001 |  |
| Non-Hispanic Black | 526/2953 | 0.966 (0.941, 0.991) | 0.008 |  |
| Other | 933/4682 | 0.981 (0.957, 1.005) | 0.111 |  |
| Education level |  |  |  | 0.037 |
| High school graduate or less | 2036/6378 | 0.979 (0.962, 0.997) | <0.05 |  |
| College or above | 1419/7337 | 0.965 (0.949, 0.982) | <0.001 |  |
| Marital status |  |  |  | 0.561 |
| Married/living with a partner | 2104/8366 | 0.967 (0.948, 0.985) | <0.001 |  |
| Divorced/separated/widowed | 1152/2855 | 0.974 (0.947, 1.002) | 0.068 |  |
| Never married | 199/2494 | 0.968 (0.930, 1.009) | 0.121 |  |
| Family PIR |  |  |  | 0.721 |
| <1.3 | 1040/4112 | 0.970 (0.949, 0.991) | 0.005 |  |
| 1.3-3.5 | 1513/5263 | 0.975 (0.954, 0.997) | <0.05 |  |
| ≥3.5 | 902/4340 | 0.965 (0.944, 0.987) | 0.002 |  |
| Noise exposure |  |  |  | 0.85 |
| Yes | 1994/6984 | 0.969 (0.953, 0.986) | <0.001 |  |
| No | 1461/6731 | 0.968 (0.951, 0.986) | <0.001 |  |
| Hypertension |  |  |  | 0.804 |
| Yes | 2210/5480 | 0.974 (0.958, 0.991) | 0.002 |  |
| No | 1245/8235 | 0.965 (0.949, 0.982) | <0.001 |  |
| Diabetes |  |  |  | 0.150 |
| Yes | 1062/2303 | 0.951 (0.921, 0.982) | 0.002 |  |
| No | 2393/11412 | 0.975 (0.962, 0.988) | <0.001 |  |
| Frequency |  |  |  |  |
| High | 5554/13696 | 0.980 (0.966, 0.993) | 0.004 |  |
| Low | 2497/13751 | 0.972 (0.958, 0.985) | <0.001 |  |
|  | | | | |
| **SF-PTA** | | | | |
| Total | 12.979 | -0.123 (-0.158,-0.089) | <0.001 |  |
| Age |  |  |  | 0.194 |
| <60 | 9.230 | -0.129 (-0.165,-0.092) | <0.001 |  |
| ≥60 | 25.041 | -0.191 (-0.308,-0.074) | 0.002 |  |
| Gender |  |  |  | 0.273 |
| Male | 14.332 | -0.128 (-0.190,-0.066) | <0.001 |  |
| Female | 11.690 | -0.113 (-0.160,-0.067) | <0.001 |  |
| Race |  |  |  | 0.138 |
| Non-Hispanic White | 13.900 | -0.135 (-0.178,-0.092) | <0.001 |  |
| Non-Hispanic Black | 10.857 | -0.114 (-0.189,-0.040) | 0.003 |  |
| Other | 10.890 | -0.060 (-0.121,-0.001) | <0.05 |  |
| Education level |  |  |  | 0.025 |
| High school graduate or less | 15.630 | -0.071 (-0.131,-0.012) | <0.05 |  |
| College or above | 11.386 | -0.146 (-0.189,-0.104) | <0.001 |  |
| Marital status |  |  |  | 0.561 |
| Married/living with a partner | 13.118 | -0.131 (-0.174,-0.087) | <0.001 |  |
| Divorced/separated/widowed | 17.992 | -0.111 (-0.209,-0.014) | <0.05 |  |
| Never married | 7.611 | -0.145 (-0.199,-0.091) | <0.001 |  |
| Family PIR |  |  |  | 0.637 |
| <1.3 | 12.991 | -0.138 (-0.208,-0.067) | <0.001 |  |
| 1.3-3.5 | 14.284 | -0.115 (-0.179,-0.050) | <0.001 |  |
| ≥3.5 | 11.912 | -0.114 (-0.172,-0.056) | <0.001 |  |
| Noise exposure |  |  |  | 0.277 |
| Yes | 13.481 | -0.096 (-0.149,-0.043) | <0.001 |  |
| No | 12.352 | -0.155 (-0.211,-0.099) | <0.001 |  |
| Hypertension |  |  |  | 0.349 |
| Yes | 18.323 | -0.128 (-0.202,-0.054) | <0.001 |  |
| No | 10.076 | -0.134 (-0.172,-0.097) | <0.001 |  |
| Diabetes |  |  |  | 0.549 |
| Yes | 20.785 | -0.084 (-0.239,0.070) | 0.283 |  |
| No | 11.818 | -0.130 (-0.166,-0.094) | <0.001 |  |
| Frequency |  |  |  |  |
| High | 20.071 | -0.130 (-0.185, -0.074) | <0.001 |  |
| Low | 10.591 | -0.114 (-0.147,-0.081) | <0.001 |  |
|  | | | | |
| **Tinnitus** | | | | |
| Total | 4706/21644 | 0.978 (0.971-0.986) | <0.001 |  |
| Age |  |  |  | 0.518 |
| <60 | 2611/13846 | 0.977 (0.967-0.986) | <0.001 |  |
| ≥60 | 2095/7798 | 0.987 (0.974-1.000) | <0.05 |  |
| Gender |  |  |  | 0.45 |
| Male | 2357/10462 | 0.985 (0.972-0.997) | <0.05 |  |
| Female | 2349/11182 | 0.973 (0.962-0.984) | <0.001 |  |
| Race |  |  |  | 0.507 |
| Non-Hispanic White | 2605/10191 | 0.979 (0.971-0.988) | <0.001 |  |
| Non-Hispanic Black | 730/4419 | 0.981 (0.963-0.999) | <0.05 |  |
| Other | 1371/7034 | 0.981 (0.961-1.001) | 0.061 |  |
| Education level |  |  |  | 0.868 |
| High school graduate or less | 2628/10926 | 0.984 (0.972-0.996) | <0.05 |  |
| College or above | 2078/10718 | 0.975 (0.963-0.987) | <0.001 |  |
| Marital status |  |  |  | 0.611 |
| Married/living with a partner | 2786/13151 | 0.967 (0.948-0.985) | <0.001 |  |
| Divorced/separated/widowed | 1264/4885 | 0.972 (0.946-0.998) | <0.05 |  |
| Never married | 656/3608 | 0.968 (0.929-1.009) | 0.125 |  |
| Family PIR |  |  |  | 0.393 |
| <1.3 | 1558/6507 | 0.968 (0.954-0.982) | <0.001 |  |
| 1.3-3.5 | 1805/8436 | 0.982 (0.968-0.996) | <0.05 |  |
| ≥3.5 | 1343/6701 | 0.982 (0.970-0.994) | 0.004 |  |
| Noise exposure |  |  |  | 0.358 |
| Yes | 2513/9607 | 0.975 (0.963-0.987) | <0.001 |  |
| No | 2193/12037 | 0.983 (0.971-0.996) | <0.05 |  |
| Hypertension |  |  |  | 0.474 |
| Yes | 2359/8878 | 0.979 (0.966-0.991) | 0.001 |  |
| No | 2347/12766 | 0.981 (0.970-0.991) | <0.001 |  |
| Diabetes |  |  |  | 0.321 |
| Yes | 908/3503 | 0.971 (0.952-0.991) | 0.004 |  |
| No | 3798/18141 | 0.980 (0.971-0.988) | <0.001 |  |

Odds ratios and regression coefficients (95% confidence intervals) were obtained after individually removing the examined variable from the weighted multivariate logistic regression models and weighted multivariate linear regression models, adjusted for age, gender, race, educational level, marital status, PIR, noise exposure, total energy intake, hypertension, and diabetes.

Abbreviations: OBS, oxidative balance score; SFHL, speech-frequency hearing loss; SF-PTA, speech-frequency pure-tone average; OR: odds ratios; β: regression coefficient; CI: confidence interval; PIR, income-to-poverty ratio

Supplementary Table S5. Sensitivity analysis of the association between OBS (tertile) and hearing loss as well as tinnitus.

|  | **Model 1** | | **Model 2** | | **Model 3** | |
| --- | --- | --- | --- | --- | --- | --- |
|  | **OR/β (95%CI)** | **P value** | **OR/β (95%CI)** | **P value** | **OR/β (95%CI)** | **P value** |
| **SFHL** | | | | | | |
| OBS (tertile) | | | | | | |
| T1 | Reference |  | Reference |  | Reference |  |
| T2 | 0.771 (0.638, 0.933) | 0.008 | 0.782 (0.639, 0.957) | ＜0.05 | 0.784 (0.640, 0.961) | ＜0.05 |
| T3 | 0.635 (0.535, 0.754) | ＜0.001 | 0.649 (0.532, 0.791) | ＜0.001 | 0.661 (0.542, 0.805) | ＜0.001 |
| p for trend | ＜0.001 |  | ＜0.001 |  | ＜0.001 |  |
|  | | | | | | |
| **SF-PTA** | | | | | | |
| OBS (tertile) | | | | | | |
| T1 | Reference |  | Reference |  | Reference |  |
| T2 | -1.234 (-1.779, -0.689) | ＜0.001 | -0.988 (-1.543, -0.432) | ＜0.001 | -0.966 (-1.522, -0.409) | ＜0.001 |
| T3 | -2.207 (-2.764, -1.650) | ＜0.001 | -1.782 (-2.366, -1.197) | ＜0.001 | -1.721 (-2.301, -1.142) | ＜0.001 |
| p for trend | ＜0.001 |  | ＜0.001 |  | ＜0.001 |  |
|  | | | | | | |
| **Tinnitus** | | | | | | |
| OBS (tertile) | | | | | | |
| T1 | Reference |  | Reference |  | Reference |  |
| T2 | 0.855 (0.768, 0.952) | 0.005 | 0.816 (0.731, 0.912) | ＜0.001 | 0.824 (0.737, 0.920) | ＜0.001 |
| T3 | 0.810 (0.719, 0.911) | ＜0.001 | 0.726 (0.628, 0.841) | ＜0.001 | 0.737 (0.637, 0.852) | ＜0.001 |
| p for trend | ＜0.001 |  | ＜0.001 |  | ＜0.001 |  |

Model 1: Adjusted for age, gender, and race.

Model 2: Combination of model 1 and educational level, marital status, PIR, noise exposure, and total energy intake.

Model 3: Combination of model 2 and hypertension, and diabetes.

Abbreviations: OBS, oxidative balance score; T, tertile; SFHL, speech-frequency hearing loss; SF-PTA, speech-frequency pure-tone average; OR: odds ratios; β: regression coefficient; CI: confidence interval; PIR, income-to-poverty ratio

Supplementary Table S6. Sensitivity analysis of the association between OBS (quintile) and hearing loss as well as tinnitus.

|  | **Model 1** | | **Model 2** | | **Model 3** | |
| --- | --- | --- | --- | --- | --- | --- |
|  | **OR/β (95%CI)** | **P value** | **OR/β (95%CI)** | **P value** | **OR/β (95%CI)** | **P value** |
| **Hearing loss** | | | | | | |
| OBS (quintile) | | | | | | |
| Q1 | Reference |  | Reference |  | Reference |  |
| Q2 | 0.769 (0.608, 0.974) | ＜0.05 | 0.792 (0.620, 1.011) | 0.061 | 0.795 (0.622, 1.016) | 0.067 |
| Q3 | 0.698 (0.542, 0.899) | 0.006 | 0.702 (0.532, 0.926) | ＜0.05 | 0.708 (0.536, 0.936) | ＜0.05 |
| Q4 | 0.653 (0.520, 0.822) | ＜0.001 | 0.647 (0.506, 0.828) | ＜0.001 | 0.653 (0.511, 0.835) | ＜0.001 |
| Q5 | 0.502 (0.391, 0.644) | ＜0.001 | 0.494 (0.371, 0.658) | ＜0.001 | 0.508 (0.383, 0.675) | ＜0.001 |
| p for trend | ＜0.001 |  | ＜0.001 |  | ＜0.001 |  |
|  | | | | | | |
| **Pure-tone average** | | | | | | |
| OBS (quintile) | | | | | | |
| Q1 | Reference |  | Reference |  | Reference |  |
| Q2 | -0.687 (-1.144, 0.070) | 0.075 | -0.360 (-1.103, 0.384) | 0.340 | -0.322 (-1.057, 0.413) | 0.387 |
| Q3 | -1.262 (-1.987, -0.538) | ＜0.001 | -0.944 (-1.694, -0.195) | ＜0.05 | -0.898 (-1.645, -0.151) | ＜0.05 |
| Q4 | -2.144 (-2.898, -1.391) | ＜0.001 | -1.711 (-2.457, -0.965) | ＜0.001 | -1.665 (-2.407, -0.923) | ＜0.001 |
| Q5 | -2.756 (-3.506, -2.006) | ＜0.001 | -2.258 (-3.066, -1.450) | ＜0.001 | -2.156 (-2.949, -1.364) | ＜0.001 |
| p for trend | ＜0.001 |  | ＜0.001 |  | ＜0.001 |  |
|  | | | | | | |
| **Tinnitus** | | | | | | |
| OBS (quintile) | | | | | | |
| Q1 | Reference |  | Reference |  | Reference |  |
| Q2 | 0.917 (0.796, 1.057) | 0.229 | 0.899 (0.779, 1.038) | 0.146 | 0.906 (0.785, 1.047) | 0.178 |
| Q3 | 0.784 (0.680, 0.904) | 0.001 | 0.732 (0.638, 0.840) | ＜0.001 | 0.742 (0.647, 0.852) | ＜0.001 |
| Q4 | 0.739 (0.626, 0.873) | ＜0.001 | 0.662 (0.563, 0.777) | ＜0.001 | 0.670 (0.570, 0.787) | ＜0.001 |
| Q5 | 0.778 (0.674, 0.898) | ＜0.001 | 0.669 (0.559, 0.801) | ＜0.001 | 0.683 (0.571, 0.818) | ＜0.001 |
| p for trend | ＜0.001 |  | ＜0.001 |  | ＜0.001 |  |

Model 1: Adjusted for age, gender, and race.

Model 2: Combination of model 1 and educational level, marital status, PIR, noise exposure, and total energy intake.

Model 3: Combination of model 2 and hypertension, and diabetes.

Abbreviations: OBS, oxidative balance score; Q, quintile; SFHL, speech-frequency hearing loss; SF-PTA, speech-frequency pure-tone average; OR: odds ratios; β: regression coefficient; CI: confidence interval; PIR, income-to-poverty ratio

Supplementary Table S7. Sensitivity analysis of the association between OBS (quartile) and hearing loss as well as tinnitus in imputed data.

|  | **Model 1** | | **Model 2** | | **Model 3** | |
| --- | --- | --- | --- | --- | --- | --- |
|  | **OR/β (95%CI)** | **P value** | **OR/β (95%CI)** | **P value** | **OR/β (95%CI)** | **P value** |
| **Hearing loss** (n=15251) | | | | | | |
| OBS (continuous) | 0.968 (0.959, 0.978) | ＜0.001 | 0.969 (0.956, 0.981) | ＜0.001 | 0.970 (0.957, 0.982) | ＜0.001 |
| OBS (quartile) | | | | | | |
| Q1 | Reference |  | Reference |  | Reference |  |
| Q2 | 0.705 (0.577, 0.861) | ＜0.001 | 0.713 (0.577, 0.881) | 0.002 | 0.713 (0.573, 0.887) | 0.003 |
| Q3 | 0.627 (0.508, 0.774) | ＜0.001 | 0.643 (0.507, 0.817) | ＜0.001 | 0.652 (0.512, 0.830) | ＜0.001 |
| Q4 | 0.511 (0.417, 0.625) | ＜0.001 | 0.506 (0.398, 0.643) | ＜0.001 | 0.509 (0.399, 0.648) | ＜0.001 |
| p for trend | ＜0.001 |  | ＜0.001 |  | ＜0.001 |  |
|  | | | | | | |
| **Pure-tone average** (n=15251) | | | | | | |
| OBS (continuous) | -0.153 (-0.184, -0.121) | ＜0.001 | -0.129 (-0.163, -0.094) | ＜0.001 | -0.123 (-0.156, -0.089) | ＜0.001 |
| OBS (quartile) | | | | | | |
| Q1 | Reference |  | Reference |  | Reference |  |
| Q2 | -1.008 (-1.695, -0.321) | 0.004 | -0.631 (-1.365, 0.102) | 0.091 | -0.582 (-1.329, 0.165) | 0.125 |
| Q3 | -2.100 (-2.706, -1.494) | ＜0.001 | -1.703 (-2.377, -1.029) | ＜0.001 | -1.611 (-2.279, -0.943) | ＜0.001 |
| Q4 | -2.942 (-3.625, -2.260) | ＜0.001 | -2.371 (-3.134, -1.608) | ＜0.001 | -2.256 (-3.023, -1.489) | ＜0.001 |
| p for trend | ＜0.001 |  | ＜0.001 |  | ＜0.001 |  |
|  | | | | | | |
| **Tinnitus** (n=24259) | | | | | | |
| OBS (continuous) | 0.988 (0.982, 0.994) | ＜0.001 | 0.978 (0.970, 0.985) | ＜0.001 | 0.978 (0.971, 0.986) | ＜0.001 |
| OBS (quartile) | | | | | | |
| Q1 | Reference |  | Reference |  | Reference |  |
| Q2 | 0.853 (0.754, 0.966) | ＜0.05 | 0.840 (0.736, 0.959) | ＜0.05 | 0.849 (0.743, 0.970) | ＜0.05 |
| Q3 | 0.792 (0.696, 0.901) | ＜0.001 | 0.713 (0.623, 0.817) | ＜0.001 | 0.725 (0.633, 0.830) | ＜0.001 |
| Q4 | 0.811 (0.715, 0.920) | 0.001 | 0.694 (0.593, 0.812) | ＜0.001 | 0.703 (0.600, 0.823) | ＜0.001 |
| p for trend | 0.001 |  | ＜0.001 |  | ＜0.001 |  |

Model 1: Adjusted for age, gender, and race.

Model 2: Combination of model 1 and educational level, marital status, PIR, noise exposure, and total energy intake.

Model 3: Combination of model 2 and hypertension, and diabetes.

Abbreviations: OBS, oxidative balance score; Q, quintile; SFHL, speech-frequency hearing loss; SF-PTA, speech-frequency pure-tone average; OR: odds ratios; β: regression coefficient; CI: confidence interval; PIR, income-to-poverty ratio
